# A comparison of polarized and non-polarized human endometrial monolayer culture systems on murine embryo development

**DOI:** 10.1186/1743-1050-2-7

**Published:** 2005-04-19

**Authors:** Mohamad Reza Baghaban Eslami Nejad, Mojtaba Rezazadeh Valojerdi, Saeed Kazemi Ashtiani

**Affiliations:** 1 Department of Embryology, Royan Institute, Tehran, Iran; 2 Department of Anatomy, School of Medical Sciences, Tarbiat Modarres University, Tehran, Iran

## Abstract

**Background:**

Co-culture of embryos with various somatic cells has been suggested as a promising approach to improve embryo development. Despite numerous reports regarding the beneficial effects of epithelial cells from the female genital tract on embryo development in a co-culture system, little is known about the effect of these cells when being cultured under a polarized condition on embryo growth. Our study evaluated the effects of in vitro polarized cells on pre-embryo development.

**Methods:**

Human endometrial tissue was obtained from uterine specimens excised at total hysterectomy performed for benign indications. Epithelial cells were promptly isolated and cultured either on extra-cellular matrix gel (ECM-Gel) coated millipore filter inserts (polarized) or plastic surfaces (non-polarized). The epithelial nature of the cells cultured on plastic was confirmed through immunohistochemistry, and polarization of cells cultured on ECM-Gel was evaluated by transmission electron microscopy (TEM). One or two-cell stage embryos of a superovulated NMRI mouse were then flushed and placed in culture with either polarized or non-polarized cells and medium alone. Development rates were determined for all embryos daily and statistically compared. At the end of the cultivation period, trophectoderm (TE) and inner cell mass (ICM) of expanded blastocysts from each group were examined microscopically.

**Results:**

Endometrial epithelial cells cultured on ECM-Gel had a highly polarized columnar shape as opposed to the flattened shape of the cells cultured on a plastic surface. The two-cell embryos cultured on a polarized monolayer had a higher developmental rate than those from the non-polarized cells. There was no statistically significant difference; still, the blastocysts from the polarized monolayer, in comparison with the non-polarized group, had a significantly higher mean cell number. The development of one-cell embryos in the polarized and non-polarized groups showed no statistically significant difference.

**Conclusion:**

Polarized cells could improve in vitro embryo development from the two-cell stage more in terms of quality (increasing blastocyst cellularity) than in terms of developmental rate.

## Background

Embryos developing in vivo are nourished by the cells and fluid of the fallopian tube and the uterine endometrium [1,2]. In vitro embryo culture deprives the cells from the maternal environment, where they are bathed in an ever-changing milieu of fluid containing a range of protein and ions specific to the reproductive process [3,4]. Although mammalian embryos can be cultured in vitro, in vivo development is superior in terms of rate of development [5-7], cell number [6], various biochemical parameters and survival following embryo transfer [8,9]. Attempts to improve in vitro culture conditions by modifying electrolyte composition and energy substrates have met with limited success [1,10]. To solve this problem and in contrast to the use of a medium alone, a number of co-culture systems mimicking the embryotrophic cell environment of the genital tract have been devised in which embryos can develop to blastocyst and hatching blastocyst stages [11-16]. Even though the beneficial effects of co-culture systems have been suggested by a number of researchers, the mechanism of action of co-culture cells has not been fully elucidated. Several investigators have proposed that co-culture systems optimize in vitro culture conditions by secreting embryotrophic substance(s) into the culture medium [17] and removing embryotoxic materials from it [18]. Embryo-feeder cell contact is also necessary to allow cellular connections between the zona and feeder cells for cross-transfer of such embryotrophic factor(s) [19]. To prepare a co-culture system, epithelial cells are usually cultivated on a plastic surface in the form of a monolayer; embryos are then cultured on it. Several studies have demonstrated that during the culture procedure performed on plastic surfaces, epithelial cells lose polarity and differentiated function (such as polarized secretion) in several days [20-23]. Furthermore, the cultivated cells lose their cubical shape, become flat and fail to keep their lateral junctions, including tight junction and desmosomes. In contrast to the loss of polarity occurring during conventional epithelial cell culture on plastic surfaces, numerous studies have demonstrated the retention of structural polarity and differentiated function when the culturing condition mimics two aspects of the in situ epithelial cell environment: baso-lateral feeding and contact with an extra-cellular matrix [24-27].

To date, several reports have shown that polarity retention of epithelial cells during culture (compared to non- polarized cells) may improve motility and fertilizing capacity of the sperm co-cultured on it [28-30]. There is little information, however, regarding co-culture of polarized cells and embryos. The aim of our investigation was to observe polarized human endometrial cells on ECM-Gel and to evaluate their effects versus those of conventional non- polarized monolayers on murine pre-embryo development.

## Methods

### Cell culture

Human endometrial tissue was obtained from patients in the secretive phase (according to full thickness of the endometrium and histological appearance of the tissue) undergoing hysterectomy at the Department of Obstetrics and Gynecology, Arash Hospital (Tehran, Iran) for benign indications. Use of the endometrial tissue for research was approved by the Ethics Committee of Royan Institute (Tehran, Iran). Tissue was excised immediately after the hysterectomy and were rinsed in Hank's balanced salt solution to remove blood and debris before being minced into small pieces of about 1 mm^3^. A small portion of each specimen was fixed in Karnovsky solution for TEM. Tissue fragments were digested by 0.25% type I collagenase in a DMEM/HAM's-F12 (Sigma, USA), containing 100 IU/ml streptomycin (Sigma, USA), 100 IU/ml Penicillin(Sigma, USA), and 10% heat inactivated fetal calf serum (FCS, Gibco, UK). After 1.5 h, the majority of glands were dissociated from surrounding tissues and floated in the digestate produced by collagenase enzyme at 37°C. The digestate also contained free stromal cells and undigested fragments. The unit gravity sedimentation technique helped isolate the endometrial glands from the other components. First, the digestate was transferred into a 13-ml plastic tube (Falcon, USA), and after its volume was reconstituted to 12 ml with DMEM/HAM's-F12, the tube was turned upside down to resuspend the digestate. The endometrial fragments were then pelleted by being allowed to settle under normal gravity for ~1 min, and the pellets containing undigested materials were subsequently discarded. The supernatants having been transferred into another tube, the glands were separated from the free cells under normal gravity, washed by centrifugation (10 min 75 g, ×2) and resuspended in a medium containing 100 IU/ml streptomycin, 100 IU/ml Penicillin, 5% FCS, 10 μg/ml epidermal growth factor (Sigma, USA) and 10 μg/ml retinoic acid (Sigma, USA). Next, they were distributed on a 12 mm diameter filter in millicell CM inserts (Millipore, USA), previously coated with ECM-Gel (Sigma, USA). Coated filters were prepared by adding 0.1 ml of 1:4 diluted ECM-Gel in DMEM/HAM's-F12 to each insert, followed by drying under a laminar flow hood. Filters were stored under sterile conditions at 4°C. The ECM-Gel coated inserts were placed in a well on a standard 24-well tissue culture plate (polystyrene plastic, Falcon, USA), containing a DMEM HAM's-F12 medium. Optimal seeding density was achieved when the glands covered approximately 30% of the surface allowing them to explant in monolayer fashion. Cultures were usually confluent after 7d. Aliquots of the gland suspension were also cultured in parallel in a well of 24-well polystyrene dish before the plates were placed in an incubator. The medium was replaced every 3 days.

### Transmission electron microscopy

The cultures on ECM-Gel and the tissue material were fixed for 1 h by Karnovsky fixative at room temperature, and then fixative was removed and the culture and tissue washed with 0.1 M OsO_4 _(Sigma, USA) in 0.1 M phosphate buffer × 1 h at 20°C. The ECM-Gel coated filter, containing glandular epithelial cells, was cut out of the millicell insert with a scalpel and subjected in parallel with the tissue material to graded ethanol dehydration before being embedded in Araldite (Araldite 2005, Sigma, USA).

Cells cultured on plastic were processed in the same manner as used for cells cultured on ECM-Gel and the tissue materials, but they were embedded differently. First, the embedding plastic blocks were filled with Araldite, and then the plastic containing the cultured cells were laid on the blocks and placed at 60°C × 18 hrs. Next, the plastic was removed and the cells remained within the resin surface in the blocks. Semi-thin sections (0.3 μm) were made and stained with toluidine blue, while the ultra-thin sections (70–100 nm) were contrasted with uranyl acetate and lead citrate and examined using a transmission electron microscope (Ziess TM 900, Germany).

### Immunohistochemistry

An immunohistochemistry staining procedure utilizing Dako Envision^+ ^system peroxidase (Dako, Denmark) was utilized in order to demonstrate the presence of cytokratin 7. The procedure was in accordance with the method proposed by the manufacturer. Briefly, the endogenous peroxidase was inhibited by incubating the cells cultured in Termanox coverslip with peroxidase block for 5 minutes, and then they were washed in distilled water and placed in wash solution. Next, the monolayer was incubated furthermore with the mouse antibody against cytokratin 7 at a concentration of 1:15 for 15 minutes. After that, it was overlaid with peroxidase labeled polymer conjugated to goat anti-mouse immunoglubins in Tris-Hcl buffer, containing carrier protein and an anti-microbial agent for 30 minutes before being washed. The last step was an incubation with DAB+ substrate- chromogen solution for 10 minutes, followed by Hematoxylin counterstaining. The negative control was monolayer undergoing stain procedure without antibody, and the positive control was endometrial frozen section. The majority (~95%) of cells isolated were epithelial.

### Embryo and co-culture specifications

Female NMRI 6–8-week-old mice underwent ovulation induction by the injection of 10 IU human menopausal gonadotropin (HMG, Organon, Holland), followed 48 h later by the injection of 10 IU human chorionic gonadotropin (HCG, Organon, Holland). Females were mated with males from the same strain. Mice with vaginal plugs were considered pregnant and sacrificed by cervical dislocation 20–22 h and 44–48 h post-hCG for one- and two-cell embryos, respectively. Embryos were flushed from the oviduct with DMEM/HAM's-F12, supplemented with 5 mg/ml bovine serum albumin (BSA, Sigma, USA). Morphologically normal embryos were washed and pooled in fresh DMEM/HAM's F12 medium before use.

Parallel to mice superovulation and embryo collection, polarized and non-polarized epithelial monolayers were prepared in 24-well plates as described above. On day 7 after initiation of culture when monolayers became confluent, the medium was replaced with DMEM/HAM's-F12, containing 5 mg/ml BSA. 24 h later, one or two-cell embryos were cultured on the monolayers. In this study, the polarized and non- polarized groups were considered as experimental group I (exp I) and experimental group II (exp II), respectively; and the medium alone was designated as the control group (con). Each co-culture experiment was replicated 5 times. Embryo developmental rate was observed every 24 hrs and recorded for 96 or 120 h. Early cleavage stage embryos were classified as degenerate if >25% of each embryo contained cytoplasmic fragments or if the blastomeres appeared dark and granular. Morula and blastocyst stage embryos were considered as degenerate if they collapsed. Finally, the results of the development were statistically compared using χ^2 ^test.

### Blastocyst differential staining

At the end of cultivation (120 hrs for one-cell and 96 hrs for two-cell embryos), expanded blastocysts of each group were randomly selected, and ICM and TE were differentially stained according to Thouas *et al. *[31]. To stain the embryos, the blastocysts were first incubated in 500 μL of solution 1 (BSA-free Hepes buffered murine tubal fluid medium + 1% triton X-100 and 100 μg/ml propidium iodide, Sigma, USA) for up to 10 sec. Blastocysts were then immediately transferred into 500 μl of solution 2 (fixative solution of 100% ethanol + 25 μg/ml bisbenzimide, Sigma, USA) and stored at 4°C overnight. Stained blastocysts were transferred from solution 2 directly into glycerol, taking care to avoid carry-over of excessive amounts of the solutions. Blastocysts were mounted on glass slides in a drop of glycerol, and cell counting was performed from images obtained from an inverted microscope fitted with an ultraviolet lamp and excitation filters (460 nm for blue and red fluorescence and 500 nm for red only). Data were analyzed by one-way analysis of variance (ANOVA).

## Results

### Cell culture

After one day of being cultured on ECM-Gel, epithelial cells grew out of the glands, spread into the intervals among the glands, seeded on ECM-Gel coated inserts, and reached confluency after 7 days. These cells, columnar in shape and packed closely together, appeared as an epithelial plate on the ECM-Gel, whereas the cells on the plastic surfaces, appeared polygonal and not packed closely together, reaching confluency after 5 days of culture.

### Polarized cell structure

**Figure 1:**
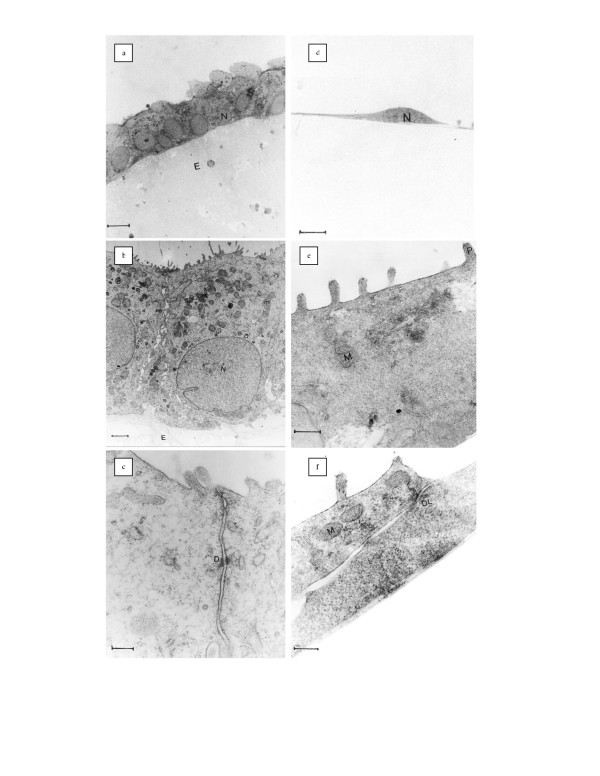
Light and Electron Micrograph of Polarized and Non-Polarized Human Endometrial Epithelial Cells in Culture. *a*: Semi-thin Section of Polarized epithelial cells;tuluidine blue staining;bar:5 μm. *b,c*: Ultra-thin Section of Polarized epithelial cells; N:nucleous; arrow: microvilli; mitochondria; jc: junctional complex; Uranyl acetate and lead citrate staining; bar b:1.6 μm; bar e: 200 nm. *d*: Semi-thin Section of non-polarized epithelial cells; Tuluidine blue staining; bar: 5 μm. *e,f*: Ultra-thin Section of non-polarized epithelial cells; arrow: Thick process; M: mitochondria; V: Vacuoles; Dl: desmosome like junction; Uranyl acetate and lead citrate staining; bar d,f: 200 nm.

The cells cultured on ECM-Gel surfaces had a highly polarized appearance with a basal nucleus and apical abundant microvilli (Figs. 1a and 1b). Lateral junctions (as desmosomes and tight junctions) were well established and basal lamina was formed under the epithelial cells (Fig. 1c). Nuclei of the in vitro cells were eukromatin with some invagination in the nuclear envelope (Fig. 1b). Their cytoplasm had abundant spherical or elongated mitochondria and cisterns of rER. In general, the cells cultured on ECM-Gel were similar to those observed in tissue.

### Non-polarized cell culture

Cells cultured on plastic surfaces (non-polarized cells) were short and spindly in section (Fig. 1d) as opposed to the columnar cells in the tissue fragment. These cells had only a few short apical processes (Fig. 1e). Extensions of adjacent cells were observed to lay upon each other and desmosome-like junctions fastened them together; there was no tight junction (Fig. 1f). The nuclei of these cells, in comparison with the epithelial cells in vivo, were relatively large. Their cytoplasm was relatively free of organelles; only a few mitochondria, cisterns of rER, and large vacuoles were seen (Fig. 1e).

### Development and blastocyst cellularity

#### One-cell embryos

A total of 287 one-cell embryos were randomly allocated to the uterine polarized group (Exp 1; *n *= 97), and uterine non-polarized group (Exp 2; *n *= 77) and DMEM/HAM's-F12 (con; *n *= 113). The rate of development and embryo degeneration in the three culture systems are shown in Additional file 1, Table 1.

Compared to the control group, embryo development was enhanced significantly by all the feeder cell cultures throughout the cultivation period (120 h). In both experiment groups, a significantly higher number of embryos reached a more advanced developmental stage (blastocysts and hatching blastocysts), compared to the control group where no blastocyst hatching was noted. Embryo development rates in the polarized group were higher than that in the non-polarized group, but the difference was not statistically significant. During the cultivation period, embryo degeneration in the polarized group was lower (without statistical difference) than that observed in the non-polarized group. However, embryo degeneration in the feeder culture groups were significantly lower than those in the control group. At the end of the cultivation period, the mean total cell number of the blastocysts developed on the feeder layers was significantly higher than that in the control group (*p *< 0.005). For this parameter there was no statistically significant difference between the polarized and non-polarized groups, but mean total cell number was higher in the polarized group. There were similar relationships between our groups in terms of the mean percentage of TE and ICM (see Additional file 2, Table 3).

#### Two-cell embryos

A total of 339 two-cell embryos were randomly allocated to the uterine polarized group (*n *= 106), uterine non-polarized group (*n *= 109) and DMEM/ HAM's-F12 (*n *= 124). Both feeder layers significantly enhanced the embryonic development; as a result, more embryos reached blastocyst and hatching blastocyst stages in the co-culture groups in comparison with those in the control group. The rate of embryo degeneration in the co-culture groups was significantly lower than that in the control. The percentage of the blastocysts and hatching blastocysts produced in the polarized group was higher than that in the non-polarized group, but the difference was not statistically significant (see Additional file 1, Table 2). Cell count results showed that the blastocysts produced in the polarized and non-polarized groups had more blastomeres than those in the control group, which was statistically significant (*p *< 0.05). Mean percentages of TE and ICM of the blastocysts from the three groups had no significant difference (see Additional file 2, Table 4).

## Discussion

Epithelial cells of the human endometrium have already been cultured on ECM-Gel for different purposes. Bentin-Ley *et al. *[32,33] used this culture system to study the changes occurring on the surface of human endometrial epithelial cells in the presence of an implanted blastocyst, while Arnold *et al. *[34] cultured these cells on ECM-Gel to describe the regulatory role of stromal cells cultured within the Gel on epithelial cell function and morphogenesis in vitro.

Park *et al. *[35] established a three-dimensional endometrial culture where human endometrial stromal cells were embedded in a mixture of collagen I and matrigel; epithelial cells were cultivated before they were replaced by KLE cells (endometrial cancer cells of epithelial origin). These investigators studied the invasion of KLE cells into the stromal fraction. Using ECM-Gel, Classen-Linke *et al. *[36] established an endometrial cell culture system to study progesterone and estrogen receptors as marker molecules for physiologically intact epithelial cells by immunohistochemistry and RT-PCR.

A number of researchers have hypothesized that polarized epithelial cells may hold promise for promoting growth and development of other cells. Pollard *et al. *suggested that maintaining cell polarity was necessary to optimize co-culture systems [37]. Having cultured epithelial cells of bovine and mouse oviducts as a polarized monolayer using Costar Transwell-Col and having also evaluated the embryo trophic potential of these cells in co-culture with mouse one-cell embryos, Ouhibi *et al. *[38] concluded that the maintenance of cell polarity of the feeder layers did not improve embryo development. It should be noted that the said authors did not use ECM-Gel for the cell polarity induction, nor did they evaluate polarity status of the cultured cells. Other investigators have cultured oviduct epithelial cells on ECM-Gel and shown that motility and fertilizing capacity of sperm is improved when co-cultured with polarized epithelial cells (compared to non-polarized cells cultured in plastic) [28-30]. Our work focused on human uterine epithelial cells that were cultured on ECM-Gel as a polarized monolayer; following polarity confirmation by TEM, their potential for improving the mouse embryo development was evaluated.

Ultra-structural evaluation of cell polarity has been the subject of previous research [22,39]. An important marker of polarity at the ultra-structural level is the presence of tight junctions at the boundary of the lateral and apical membrane domains. Vega Salas *er al. *[40] suggested that the interaction between ECM and epithelial cells generated differences in protein distributions between contacting and non-contacting surfaces and that these interactions also refined the apical-basal polarity, the useful sign of which is the location of tight junction at the apical-lateral membrane boundary. This junction prevents the mixing of specific proteins from different membrane domains. Other signs of cell polarity at the ultra-structural level include the reduction of distance between two adjacent cells, basal location of nuclei, and formation of apical microvilli. Our ultra-structural images showed that the cells cultured on ECM-Gel were polar and quite similar to cells observed in vivo.

Selecting a medium which could support both embryo and feeder cells presented the main challenge in our study. Frasor *et al. *[12] examined growth and development of mouse two-cell embryos on human oviduct epithelial cells using HTF (a simple medium able to support embryo development) and MEM-α (a complex medium capable of supporting somatic cell growth) and concluded that optimizing of growth characteristics of somatic cells in culture would improve embryo development. Several media were used in this research so that a polarized monolayer could be established; nonetheless, DMEM/HAM's-F12 was used as the co-culture medium as it was the only medium supporting the growth and maintenance of the polarized cells. DMEM/HAM's-F12 has also been used by other investigators as a co-culture medium. Specifically, Freeman *et al. *[41] cultured two cell mouse embryos on fibroblast, oviduct, and uterine epithelial cells as well as on follicular cells using DMEM/HAM's- F12 medium. It is noteworthy that their blastocyst rates were slightly higher than those in our study, although this could be associated with different mouse strains used in each experiment (B_6_C_3_F_1 _versus NMRI).

Our investigation showed that one-cell embryos of the polarized and non-polarized groups developed statistically well compared with those of the control, but the polarized groups showed no advantage over the non-polarized group when it came to improving the in vitro development of embryos from one-cell stage. This may be because of a developmental block occurring at the two cell stage, or perhaps the embryotrophic effects of the polarized monolayer manifest only when the co-culture system is initiated by using two-cell stage embryos. It seems that in co-culture systems, the activation mechanism is insufficient to overcome the embryo developmental block. This may cause the embryo not to respond properly even to positive influence of the polarized group. Such embryos would be expected to have a lower cleavage rate and reduced cellularity at the blastocyst stage, compared to those co-cultured from the 2-cell stage.

We also found a similar proportions of TE and ICM in the blastocysts that originated from both one- and two-cell embryos. It seems that irrespective of total cell number, cell allocation mechanisms put specified proportions of the cells into the TE and ICM component of each blastocyst. Indeed, simillar results were reported by Sherban *et al *[12].

One positive effect of a polarized culture system on two-cell embryo development would be reflected as efficiency of blastocyst production. Our results demonstrate that blastocysts developing from embryos cultured in a polarized state acquire significantly more blastomeres compared with those cultured among non-polarized cells. High mean cell numbers are also indicative of higher early cleavage rates and cell viability. In addition, enhanced embryo implantation may result from an increased rate of blastocoel fluid production and earlier attainment of a critical hatching diameter [42]; increased production of zona lysine by TE cells must also be considered [43].

Handyside and Hunter [44] demonstrated that the proportion of TE increased from ~60% in early mouse blastocysts to ~83% before implantation. In the first 36 h after blastocyst formation, TE components increase exponentially and then plateau as the increase in ICM cell numbers slows; this is coincident with the period of apoptosis in the ICM [45]. These influences result in an increase in the proportion of TE cells in the blastocyst during the period of blastocyst expansion and hatching. Our observations in this regard are consistent with those reported previously by Sherban *et al. *[46]. From this it may be hypothesized that blastocysts with a greater relative TE cell proportion are more advanced than those with a low TE cell proportion. In the present study, cell counting was performed at the end of a 120 h culture interval. Our results indicate that the mean TE percentage of embryos cultured on polarized cells is higher than that of embryos cultured on on-polarized cells, although the difference did not reach statistical significance.

It may be that the embryotrophic effects of experiment I (polarized group) are due both to the ECM and the polarized cells themselves, but it should be noted that cells cultured on ECM-Gel completely occupy all available surfaces of ECM-Gel and furthermore tight junctions retain ECM-Gel in the lower compartment during co-culture. This had the effect of rendering no exposed surfaces of ECM-Gel to influence the embryo development and therefore all embryotrophic effects would be due to highly polarized columnar epithelial cells. Positive influences of polarized cells on embryo growth and blastocyst cellularity produced from two-cell embryo development may be due to their differentiated state and morphology on matrigel. Given that feeder cells provide their effect by secreting an embryotrophic agent, it is logical to speculate that polarized cells by releasing different embryotrophic materials into the culture medium can improve embryo quality more than their non-polarized counterparts. Thomas *et al. *[47] found that oviduct epithelial cells polarized in vitro, had a different protein composition compared to those of the plastic cultured non-polarized cells. Also, Wolddesenbet and Newton [48] indicated that oviduct epithelial cells in polarized cultured had similar secretions to those of the cultured tissue fragment. The effect of the direct contact between feeder cells and embryos on embryo development must also be considered; preventing such contact reduces the positive effect of co-culture cells on the embryo. In polarized co-culture systems, feeder cells are columnar in shape and lie very close to one another, so each embryo establishes direct contact with more cells and probably gets more positive influences compared to those on the non-polarized cells. Determining the precise composition of the polarized cell secretions and the mechanism of action of direct contact on embryo development in co-culture systems requires more investigation.

## Conclusion

Our results suggest that epithelial cells of human oviduct, being cultured in a polarized condition, could improve blastocyst quality when co-cultured with two-cell embryos (but not one-cell embryos). It seems that providing an appropriate environment capable of supporting feeder layer as well as embryo growth would further improve in vitro development of one and two-cell mouse embryos in terms of both blastocyst formation rate and quality.

## Supplementary Material

Additional File 1 file containing table 1 and 2 Click here for file

Additional File 2 file containing table 3 and 4 Click here for file

## References

[B1] Quinn p, Kerin JF, Warnes GM (1985). Improved pregnancy rate in human in vitro fertilization with the use of a medium based on the composition of human tubal fluid. Fertil Steril.

[B2] Tervit HR, Whittingham DG, Rowson LEA (1972). Successful culture in vitro of sheep and cattle ova. J Reprod Fertil.

[B3] Neider GL, Macon GR (1987). Uterine and oviductal protein secretion during early pregnancy in the mouse. J Reprod Fert.

[B4] Leese HJ (1988). The formation and function of oviduct fluid. J Reprod Fert.

[B5] Bowman P, McLaven A (1970). Cleavage rate of mouse embryos in vivo and in vitro. J Embryol Exp Morphol.

[B6] Harlow GM, Quinn P (1982). Development of preimplantation mouse embryo in vivo and in vitro. Aust J Biol Sci.

[B7] Erbach GT, Lawitts JA, Papaioannou VE, Biggers JD (1994). Differential growth of the mouse premplantation embryo in chemically defined media. Biol Reprod.

[B8] Jung T, Fisher B (1988). Correlation between diameter and DNA or protein synthetic activity in rabbit blastocyst. Biol Reprod.

[B9] Carney EW, Foote RH (1990). Effects of super ovulation, embryo recovery, culture system and embryo transfer on development of rabbit embryos in vivo and in vitro. J Reprod Ferrtil.

[B10] Fitzgerald L, Di Mattina M (1992). Improved medium for long-term culture of human embryos overcomes the in vitro developmental block and increase blastocyst formation. Fertil steril.

[B11] Barmat LI, Worrilow KC, Payton BV (1997). Growth factor expression by human oviduct and buffalo rat liver co-culture cells. Fertil Steril.

[B12] Frasor J, Sherbahn R, Soltes B, Molo MW, Binor Z, Radwanska E, Rawlins R (1996). Animal experimentation: optimizing tubal epithelial cell growth promotes mouse embryo hatching in co- culture. J Assit Reprod Genet.

[B13] Hoshi K, Kanno Y, Katayose H, Yanagida K, Suzuki R, Sato A (1994). Coculture of mouse embryo with cryopreserved human oviduct epithelial cells. J Assist Reprod Genet.

[B14] Gandolfi F, Moor RM (1987). Stimulation of early embryonic development in the sheep by co-culture with oviduct epithelial cells. J Reprod Fert.

[B15] Sakkas D, Trounson AO, Kola I (1989). In vivo cleavage rates and viability obtained for early cleavage mouse embryo in co-culture with oviduct cells. Roprod Fertil Dev.

[B16] Lavranos TC, Seamark RF (1989). Addition of steroid to embryo-uterine monolayer co-culture enhances embryo survival and implantation in vitro. Reprod Fertil Dev.

[B17] Thibodeaux J, Godke R (1992). In vitro enhancement of early stage embryos with co-culture. Arch Pathol Lab Med.

[B18] Fukui Y, McGowan LT, James RW, Pugh PA, Tervit HR (1991). Factors affecting the in vitro development of blastocysts of bovine oocytes matured and fertilized in vitro. J Reprod Fertil.

[B19] Bongso A, Soon-Chye NG, Chui-Yee Fong, Shan Ratnam (1991). Co-cultures: a new lead in embryo quality improvement for assisted reproduction. Fertil Steril.

[B20] Glasser SR, Julian J, Decker GL, Tang JP, Carsen DD (1988). Development of morphological and functional polarity in primary cultures of immature rat uterine epithelial cells. J Cell Biol.

[B21] Jacobs AL, Decker GL, Glasser SR, Julian J, Carsen DD (1990). Vectorial secretion of prostaglandins by polarized rodent uterine epithelial cells. Endocrinology.

[B22] Schatz F, Gordon RE, Lanfer N, Gurpide E (1990). Culture of human endometiral cells under polarizing conditions. Differentiation.

[B23] Mani SK, Decker GL, Glasser SR (1991). Hormonal Responsiveness by immature rabbit uterine epithelial cells polarized in vitro. Endocrinology.

[B24] Ailenberg M, Fritz IB (1988). Control of levels of plasminogen activator activity secreted by sertoli cells maintained in a two-chamber assembly. Endocrinol.

[B25] Carson DD, Tang J-P, Julian J, Glasser SR (1988). Vectorial secretion of proteoglycans by polarized rat uterine epithelial cells. J Cell Biol.

[B26] Chembard M, Verrier B, Gabrion J, Mauchamp J (1983). Polarization of thyroid cells in culture: evidence for basolateral localization of the iodide "pump" and at the thyroid stimulating hormone receptor- adenly cyclase complex. J Cell Biol.

[B27] Hardley MA, Byers SW, Snarez-Quarez-Quian CA, Kleinman HA, Dym M (1985). Extracellular matrix regulates sertoli cell differentiation, testicular cord formation, and germ cell development in vitro. J Cell Biol.

[B28] Pollard JW, Plante C, King WT, Hansen PJ, Betteridge KJ, Suarez SS (1991). Fertilizing capacity of bovine sperm may be maintained by binding of oviductal epithelial cells. Biol Reprod.

[B29] Sidhu KS, Mate KE, Rodger JC (1988). Sperm – oviduct epithelial cell monolayer co-culture: an in vitro model of sperm female tract interactions in a marsupial, the tamer wallaby (macropus engenii). J Reprod Fertil.

[B30] Ellington JE, Jones AE, Davitt CM, Schneider CS, Brishois RS, Hiss GA, Wright RW (1998). Human sperm function in co-culture with human, macaque or bovine oviduct epithelial cells monolayers. Hum Reprod.

[B31] Thouas GA, Korfiatis NA, French AJ, Jones GM, Trounson AO (2001). Simplified technique for differential staining of inner cell mass and trophoectoderm cells of mouse and bovine blastocysts. Reprod Biomed Online.

[B32] Bentin-Ley U, Horn T, Sjogren A, Sorensen S, Falck Larsen J, Hamberger L (2000). Ultra structure of human blastocyst – endometrial interactions in vitro. J Reprod Fertil.

[B33] Bentin-Ley U, Sjogren, Nilsson L, Hamberger L, Larsen JF, Horn T (1999). Presence of uterine pinopodes at the embryo – endometrial interface during human implantation. In vitro hum Reprod.

[B34] Arnold JT, Kaufman DG, Seppala M, Lessey BA (2001). Endometrial stromal cells regulate epithelial cell growth in vitro: a new co-culture model. Hum Reprod.

[B35] Park DW, Choi DS, Ryu HS, Know HC, Joo H, Min CK (2003). A well – defined in vitro three dimensional culture of human endometrium and its applicability to endometrial cancer invasion. Cancer Lett.

[B36] Classen-Linke I, Kusche M, Knauthe R, Beier HM (1997). Establishment of a human endometrial cell culture system and characterization of its polarized hormone responsive epithelial cells. Cell Tissue Res.

[B37] Pollard JW, Xu KP, Porie R, King WA, Betteridge KJ (1989). Influence of various oviductal epithelial cell culture systems on the development of early cleavage stage bovine embryos in vitro. Theriogenology.

[B38] Ouhibi N, Hamidi J, Guillad J, Menezo Y (1990). Co-culture of one cell mouse embryo on different cell support. Hum Reprod.

[B39] Bentin-Ley U, Pederson B, Linderberg S, Larsen JB, Hamberger L, Horn T (1994). Isolation and culture of human endometrial cells in a three – dimensional culture system. J Reprod Fertil.

[B40] Vega Salas DE, salas PJ, Gunderson D, Rodriguez-Boulan E (1987). Formation of the apical pole of epithelial (madin – Darby Canine Kidney) cells : Polarity of an apical protein is independent of tight junctions while segregation of a basolateral marker requires cell – cell interactions. J Cell Biol.

[B41] Freeman MR, Bastias MC, Hill GA, Osteen KG (1993). Co- culture of mouse embryos with cells isolated from the human ovarian follicle, oviduct and uterine endometrium. Fertil Steril.

[B42] Boatman DE, Bavister BD (1987). In vitro growth of non-human primate pre- and peri-implantation embryos:Mammalian Preimplantation Embryo.

[B43] Schiewe MC, Hazeleger NI, Sclimenti C, Balmaceda JP (1995). Physiological characterization of blastocyst hatching mechanisms by use of a mouse antihatching model. Fertil Steril.

[B44] Handyside AH, Hunter S (1986). Cell division and death in the mouse blastocyst before implantation. Roux's Arech Dev Biol.

[B45] Hardy k, Handyside AH, Winston RM (1989). The human blastocyst:cell number, death and allocation during late preimplantation development in vitro. Development.

[B46] Sherban R, Frasor J, Radwanska E, Binor Z, Wood-molo M, Hibner M, Mack S, Rawlines RG (1996). Comparison of mouse embryo development in open and micro drop Co- Culture systems. Hum Reprod.

[B47] Thomas PG, Ignotz GG, Ball BA, Miller PG, Brinsko SP, Currie B (1995). Isolation, Culture and characterization of equine oviduct epithelial cells in vitro. Mol Reprod Dev.

[B48] Woldesenbet S, Newton GR (2003). Comparison of proteins synthesized by polarized caprine oviductal epithelial cells and oviductal explants in vitro. Theriogenology.

