# Supplementary material for: A comparison of polarized and non-polarized human endometrial monolayer culture systems on murine embryo development
Source: J Exp Clin Assist Reprod. 2005 Apr 19;2:7. doi: 10.1186/1743-1050-2-7 (PMC1097752; doi:10.1186/1743-1050-2-7)
Supplement: Additional File 1 — file containing table 1 and 2 [file 1743-1050-2-7-S1.doc]

Table 1: Mouse one cell embryo development and degeneration in the uterine co-culture groups and control

| *Group* | *Total embryos* | *24h* | *48h* | | | *72h* | | | *96h* | | | *120h* | | |
| --- | --- | --- | --- | --- | --- | --- | --- | --- | --- | --- | --- | --- | --- | --- |
|  |  | *2 cell Embryos* | *4-8 cell Embryos* | *Morulla* | *Degenerate Embryos* | *4-8 cell Embryo* | *Morrula+ Blastocysts* | *Degenerate Embryos* | *Blastocysts* | *Hatching Blastocysts* | *Degenerate Embryos** | Blastocysts | Hatching Blastocysts | *Degenerate Embryos** |
| Control | 113 | 95  (84.07) | 51  (45.13) | 15  (13.27) | 3  (2.65) | 53  (46.90) | 14  (12.38) | 7  (6.19) | 12  (10.61) | 0  (0) | 78  (69.02) | 17  (15.04) | 0  (0) | 96  (84.95) |
| Exp I | 97 | 89  (91.75) | 66^b^  (68.04) | 8  (8.24) | 1  (1.03) | 36  (37.11) | 40^b^  (41.23) | 4  (4.12) | 21^a^  (21.46) | 5^a^  (5.15) | 50^a^  (51.54) | 18  (18.55) | 12^b^  (12.37) | 67^a^  (69.07) |
| Exp II | 77 | 66  (85.71) | 50^c^  (64.93) | 6  (7.79) | 1  (1.29) | 27  (35.06) | 30^d^  (38.96) | 4  (5.19) | 17^c^  (22.07) | 2  (2.59) | 44  (57.14) | 13  (16.88) | 8^d^  (10.38) | 56^c^  (72.72) |

* : In these times of cultivation , the degenerate rate was mostly due to colapsinization of the blastocyst.

Note: The number within parenthesis is stated in percentage. Control: DMEM/Ham’s- F12 Medium; Exp I: Polarized monolayer; Exp II: Non- polarized monolayer.

a: Exp I versus control, P<0.005; b: Exp II versus control, P<0.001; c: Exp II versus control, P<0.05; d: Exp II versus control, P<0.001

Table 2:Mouse two cell embryo development and degeneration in the uterine co-culture groups and control

| *Groups* | *Total Embryos* | *24h* | | | *48h* | | | *72h* | | | *96h* | | |
| --- | --- | --- | --- | --- | --- | --- | --- | --- | --- | --- | --- | --- | --- |
|  |  | *4-8 cell* | *Morulla* | *Degenerate embryos* | *Morrula* | *Blastocyst* | *Degenerate embryos* | *Blastocysts* | *Hatching*  *Blastocysts* | *Degenerate embryos** | *Blastocysts* | *Hatching*  *Blastocysts* | *Degenerate embryos** |
| Control | 124 | 72  (58.06) | 21  (16.93) | 0  (0) | 43  (34.67) | 18  (14.51) | 27  (21.77) | 33  (26.61) | 8  (6.45) | 56  (45.16) | 20  (16.12) | 15  (12.09) | 89  (71.77) |
| Exp I | 106 | 71  (66.98) | 35^a^  (33.01) | 0  (0) | 55^a^  (51.88) | 23  (21.69) | 13  (12.26) | 56^b^  (52.83) | 19^a^  (17.92) | 24^b^  (22.64) | 44^b^  (41.50) | 32^b^  (30.18) | 30^b^  (28.30) |
| Exp II | 109 | 68  (62.38) | 32^c^  (29.35) | 0  (0) | 54^c^  (49.54) | 22  (20.18) | 16  (14.67) | 52^d^  (47.70) | 17^c^  (15.59) | 31^c^  (28.44) | 39^d^  (35.77) | 30^c^  (27.52) | 40^d^  (36.69) |

* : In these times of cultivation , the degenerate rate was mostly due to colapsinization of the blastocyst.

Note: The number with in parenthesis is stated in percentage; Control: DMEM/Ham’s F12 Medium; Exp I: Polarized monolayer; Exp II: Non polarized monolayer.

a: Exp I versus control (P<0.05); b: Exp II versus control (P<0.001); c: Exp II versus control (P<0.05); d: Exp II versus control (P<0.001)
