# Supplementary material for: A comparison of polarized and non-polarized human endometrial monolayer culture systems on murine embryo development
Source: J Exp Clin Assist Reprod. 2005 Apr 19;2:7. doi: 10.1186/1743-1050-2-7 (PMC1097752; doi:10.1186/1743-1050-2-7)
Supplement: Additional File 2 — file containing table 3 and 4 [file 1743-1050-2-7-S2.doc]

#### **Table 3:The Cell count result of the blastocyst produced from the one cell mouse** embryo development

| Group | Total Embryo | Total Mean | TE Mean  Percentage | ICM Mean  percentage |
| --- | --- | --- | --- | --- |
| Control | 10 | 35.44±17.01 | 78.34±4 | 21.66±4 |
| Exp I | 10 | 57.10±8.56^a^ | 80.69±2.36 | 19.31±2.36 |
| Exp II | 9 | 54.22±13.59^b^ | 79.34±3.04 | 20.66±3.04 |

Note:Control: DMEM/Ham’s F12 Medium; Exp I: Polarized monolayer; Exp II: Non-polarized monolayer;TE:trophoectoderm; ICM:inner cell mass.

a: Exp I versus control ,P<0.005; b: Exp II versus control ,P<0.005

#### **Table 4:The Cell count result of the blastocyst produced from the two cell mouse** embryo development

| Group | Total Embryo | Total Mean | TE Mean Percentages | ICM Mean percentage |
| --- | --- | --- | --- | --- |
| Control | 15 | 70.46±19.01 | 79.86±3.93 | 20.14±3.93 |
| Exp I | 14 | 118.25±28.39^a,c^ | 81.36±2.18 | 18.64±2.18 |
| Exp II | 15 | 95.80±24.32^b^ | 80.48±3.17 | 19.52±3.17 |

Note:Control: DMEM/Ham’s F12 Medium; Exp I: Polarized monolayer; Exp II: Non-polarized monolayer

a: Exp I versus control ,P<0.001; b: Exp II versus control ,P<0.05; c: Exp I versus II, P<0.05
